# The importance of school leaders in school health promotion. A European call for systematic integration of health in professional development

**DOI:** 10.3389/fpubh.2023.1297970

**Published:** 2024-01-04

**Authors:** Karina Leksy, Grzegorz Gawron, Rafaela Rosário, Marjorita Sormunen, Veronica Velasco, Anita Sandmeier, Venka Simovska, Tomasz Wojtasik, Kevin Dadaczynski

**Affiliations:** ^1^Department of Social Science, Institute of Pedagogy, University of Silesia, Katowice, Poland; ^2^Department of Social Science, Institute of Sociology, University of Silesia, Katowice, Poland; ^3^School of Nursing, University of Minho, Braga, Portugal; ^4^Health Sciences Research Unit: Nursing (UICISA: E), Nursing School of Coimbra, Braga, Portugal; ^5^Research Centre in Nursing, School of Nursing, University of Minho, Braga, Portugal; ^6^Institute of Public Health and Clinical Nutrition, University of Eastern Finland, Kuopio, Finland; ^7^Department of Psychology, University of Milano-Bicocca, Milan, Italy; ^8^Schwyz University of Teacher Education, Goldau, Switzerland; ^9^Danish School of Education, Aarhus University, Copenhagen, Denmark; ^10^Regional In-Service Teachers Training Centre Metis in Katowice, Katowice, Poland; ^11^Department of Health Sciences, Fulda University of Applied Science, Fulda, Germany; ^12^Center for Applied Health Science, Leuphana University Lueneburg, Lueneburg, Germany

**Keywords:** school principals, health-promoting leadership, pre-service and in-service training, competence framework, Health Promoting School

## Abstract

School leaders such as principals are key not only for educational and school quality but also for initiating and sustainably anchoring any innovation in schools. Although there is widespread agreement about the importance of holistic approaches to school health promotion, the role of principals has received increased, but not yet systematic, attention. In this context, it is unclear which leadership competencies are needed and to what extent they are taught. Hence, this paper aims to reflect whether and to what extend health promotion plays a role in preservice and inservice training of principals in Europe. Based on the results we call for a more systematic analysis of existing teaching opportunities and teaching needs for health-promoting leadership, the development of a European competence framework for health-promoting leadership, the development and implementation of a European system that monitors and evaluates the effectiveness of those preservice and inservice training programs, and the development of case-studies to stimulate a mutual learning process.

## Introduction

### School principals in school health promotion

School leaders matter for many essential school outcomes, including student achievements, positive school climate, and productive teacher work. However, the size of this influence differs (1) and depends on individual, organizational, and system factors. Grissom et al. argue that effective school principals should orient their practice toward constructive interactions with teachers, build a positive school climate, facilitate collaboration and professional learning, and have strategic personnel and resource management processes (1). School leaders play a primary role in decision-making, meaning their decision is “a critical component of organizational actions” (2). In line with this, school principals are also crucial for co-creating and supporting a healthy environment for the whole school community.

Undoubtedly, during the last decades schools have been recognized as a key setting for health promotion. Due to compulsory schooling, a large proportion of children and young people, regardless of their social, economic and cultural background, can be easily reached through school based interventions. Hence, schools provide a window of opportunity to promote health behaviors and health as early as possible in the lifespan (3). This is especially important as research increasingly show that mental and physical health indicators are also related to academic performance, achievement, and school outcomes which highlights health to be a driving force for educational success (4–7). In this context school principals, play an important role in initiating, implementing, and sustaining health promotion and education efforts across the curriculum and integrating them into the school organization and culture. However, research has shown that although school principals view the school as an important arena for health promotion, their support for related school practices lags behind (8, 9). In times of (neo-liberal) school reforms based on different, often conflicting educational principles and approaches, the issue is how much room school principals truly have to facilitate and lead these school and curricular development processes. Moreover, the scope of action of principals depends on various aspects of educational governance, including the political and institutional anchoring of health promotion (10), but also on the available resources, school autonomy, accountability, market orientation, and the influence of other actors and networks (11).

Given their importance, school principals must be equipped with the skills and resources they need to take responsibility for promoting health in their schools. The following perspective aims to shed light on these skills and abilities by raising the question, wether and how health issues are addressed in initial and in-service training of school principals in Europe.

## The multiple facets of health-promoting leadership

In many countries, the role of the school principals has undergone intensive changes over the last couple of decades. In today's rapidly changing world, schools face a variety of challenges, which include amongst others the increasing quantity and quality of health information (known as the infodemic), the pressing issue of climate change, or political and economic insecurities. These challenges are not unrelated to health and have led to an increase in health-related problems, not least as a result of the COVID-19 pandemic (12–15). These complex and interconnected challenges necessitate educational leaders who can navigate and address them effectively by also advocating a positive approach of health and wellbeing. This requires a health-promoting leadership which has been defined “[...] as leadership that is concerned with creating a culture for health-promoting workplaces and values to inspire and motivate the employees to participate in such a development” (16). While the importance of health-promoting leadership in the school setting has been emphasized for quite some time (17, 18) there has been little discussion of what kind of skills should be included here. So far, competencies necessary for health education and health promotion in schools have been discussed specifically for teachers (19–21). While Mikkonen et al. (20) and Moynihan et al. (21) identified knowledge, skills and attitudes as core competences of health education and science, Carlsson (19) synthesized five domains of professional competencies in school health promotion (policy development, organizational development, professional development, development of students' learning, development of health promoting activities). In their recent special issue, Dadaczynski et al. (22) suggested three perspectives of leadership in school health promotion:

- **Self-related health-promoting leadership** concerns the way school principals deal with their own health, especially in light of high professional demands. Increasing evidence show that school principals suffer from high workload and work stress that rised during the COVID-19 pandemic and that is associated with mental health problems (23–26). With increasing work demands, the likelihood of maladaptive coping strategies (e.g., extensification and intensification of work) increases, which in turn can have a detrimental effect on indicators of mental health (24, 25). Considerably less research focuses on deputy principals or members of the school management team. Although inconclusive, study findings from Asia show that deputy principals perceived more stress during the pandemic (25) and were more often affected by symptoms of depression (27). This may be due to the role ambiguities and high workloads to which deputy heads are exposed (e.g., higher teaching duties and leadership tasks). Promoting their health would be equally important, as deputy principals are future principals and health problems could lead them to leave this career path prematurely. Self-centered health-promoting leadership therefore requires school leaders to be able to adequately assess their workloads and demands and to take self-care for the maintenance and promotion of their own health.
- **Staff-related health-promoting leadership** includes activities toward health care and support of teaching and non-teaching school staff. Although research in the school setting is scarce, findings indicate that principal support and specific leadership styles such as transformative or salutogenetic leadership are associated with teacher satisfaction and indicators of mental halth (28– 30). Principals who pursue a staff-focused health-promoting leadership style demonstrate a high level of interest in the current work situation and its influence on the satisfaction and wellbeing of teaching and non-teaching school staff. They take into account the needs and abilities of the employees and align them with the expectations and work demands. In addition, they enable regular exchange within the whole school team and support systematic cooperation and mutual support.
- **Intervention-related health-promoting leadership** is concerned with activities and management practices that need to be taken to support health-promoting change processes in the school (17, 31, 32). Findings from a very recently published systematic literature review revealed e.g., that principals's attitudes, beliefs, knowledge, general involvement and support were important aspects of implementing activities on school health promotion (32). Indeed, findings from Germany show that positive attitudes toward the Health Promoting School (HPS), their perceived competencies to support HPS implementation, but also their decision latitude and their perception that health promotion result in educational benefits were positivelely associated with the actual implementation status (33, 34). Moreover, their ability to find, understand, critically evaluate and apply health information (i.e., health literacy) was linked with HPS implementation, which in another study from Switzerland was moderated by positive attitudes toward HPS (35). Particularly in the field of educational research, the specific styles and factors underlying successful leadership are being discussed. With regard to educational leadership research, there is much discussion about the specific styles or factors that underlie successful leadership and how these are linked to desirable school outcomes (36). Research suggests that a visonary, i.e., a transformative leadership style is associated with a positive and innovation-friendly school culture as perceived by teachers (37, 38). However, it is important to emphasize that leadership directed toward health-promoting or educational change processes is not limited to principals alone. Depending on varying educational policies und strcutures in each country, this may also include mid-level management and specialized health managers. As highlighted by Samdal and Rowling (39) and others, successful change process are based on a balance of leadership and management practices. This requires distributed leadership, a leadership practice in which responsibilities are shared across many shoulders and groups and which can have beneficial effects on school improvement (40).

As emphasized by Dadaczynski et al. (22) these three dimensions are highly interconnected and influenced by political and infrastructural conditions at the school, local and national levels.

## School principals' professional development in health promotion

In most European countries, school principals start their educational career as teacher with main focus on teaching. There is widespread agreement that the transition from teacher to school principal or deputy principals, however, requires specialized skills due to e.g., decentralization, i.e., shifting responsibilities and power to the school level, increasing complexity of school tasks and contexts as well as the expansion of the roles and tasks of school principals (41, 42). Hence, principalship is seen as an occupation in itself which demands continuous professional development and skill acquisition. Findings from the OECD Teaching and Learning International Survey (TALIS) show that 85% of teachers completed a training or educational programme before starting their principalship, while 5% received training in teaching after becoming a school principal and 10% had no preparation at all (43).

As pointed out by Sahlin (42), unlike preservice training, largely little is known about the professional development of school leaders with work experience (in-service training).

Although school health promotion and prevention have been high on the agenda of Public Health research, practice and policy for many years, it is not yet known whether and to what extent prospective and experienced school leaders are prepared with regard to the three dimensions on health-promoting leadership outlined above. Without claiming to be exhaustive, we have conducted an initial screening for six European countries (Germany, Poland, Finland, Italy, Portugal, and Switzerland) to determine whether health promotion and prevention play a role in the preservice and inservice training of school principals.

Regarding the structure and organization of initial training and in-service training for school principals, there is a great deal of diversity among and within the countries. Due to the federal structure, the qualification of school principals is not regulated nationwide, but at the level of the federal states in Germany. All except two German federal states require mandatory training for prospective school principals, which takes between 5 and 37 days in total (44). In Switzerland, training for principals are organized in a decentralized manner by teacher training colleges in the German speaking part and more centralized in French-speaking Switzerland. In some countries, specialized Master programmes on advanced studies (Switzerland), management in education (Poland), or school administration and management (Portugal). The same situation applies to in-service training for school principals that is often regulated on a national or federal level but organized by regional or local stakeholders. In Italy, principals have no obligation to undergo in-service training. However, a wide range of trainings are offered on a regional level that also includes activities provided by the Health Promoting School network. While in Switzerland there are common standards for the further training of school principals, the landscape in Germany is very divers with training activities provided by variety of stakeholders at the federal state level (45). For Finland, the Ministry of Education and Culture has defined six focus areas for educational training that do not directly address health promotion but are indirectly related to health (e.g., Promoting welfare, safety and social skills in learning communities, promoting a sustainable lifestyle, equality, and non-discrimination). Concrete educational training activities are organized by the Finnish Regional State Administrative Agencies, but several other in-service training providers and content for principals are also available. A similar situation is found in Portugal, where in-service training encompasses diverse areas of knowledge that are relevant to teaching and educational practice and that are approved and accredited by a National Council.

Comparing the current situation across the six European countries, it becomes clear that health promotion and prevention are not systematically anchored in the initial and in-service training of school principals. With regard to **self-related health-promoting leadership** there are several further training offers available in only a few German federal states that focus on mental health-related topics such as stress and coping and are voluntary. The same can be seen in Poland with emphasis on stress management and burnout prevention but also on broader topics of self-management and development. All other countries do not explicitly address health topics on an individual level, but stress individual skills in a more generic perspective (e.g., self- and time-management, professional identity as a school leader). In Portugal, initial-training includes determinants of healthy lifestyles such as physical activity which however aims at school children. Even more rarely addressed are topics of the **staff-related health-promoting leadership** dimension. While for Finland and Portugal no specific activities could be found, for all other countries health is not directly mentioned in available training documents and catalogs, but implicitly addressed through a number of activities that focus more broadly on staff and team development, communication and feedback skills or social support provision. Only in two out of 16 German federal states healthy leadership or salutogenic leadership is included in voluntary training offers with the last approach focusing on the three dimensions of the sense of coherence concept (comprehensibility, manageability and meaningfulness) and how they can be implemented in information and communication processes of the school (46). Finally, aspects on **intervention-related health-promoting leadership** (e.g., development of health promotion policies and organizations) are not explicitly addressed in most of the countries examined. As for the other dimensions, although health is not mentioned directly, the issues addressed here have implicit relevance for school health promotion. These include general change management, project development, quality development and management or cooperation with parents and the surrounding school community. For Switzerland, the Schwyz University of Teacher Education explicitly addresses the importance of health in the organization of work, while in two German federal states training offers cover issues on health management and healthy school climate. Moreover, in some countries (e.g., Italy, Finland) school principals are supported in developing a strategic action plan on school improvement and development and in setting up start-up projects.

## A need for action: concluding remarks

Worldwide, the educational system has undergone a substantial crisis during recent years. In addition to the COVID-19 pandemic, other crises include climate change, war, economic and energy crises, and the related refugee movement. All these crises are associated with immense educational and also health-related costs, not only for children and adolescents, but also for teaching and non-teaching school staff. For school staff in particular, these crises result in new demands that are often accompanied by work stress and increase the likelihood of long lasting health problems (14, 23, 24). On the one hand, the health of teachers is related to the quality of instruction and schooling (47), and it is to be expected that the increase in health-related stress and strain will further reduce the attractiveness of the teaching profession. For years, there has been a worldwide shortage of teachers and school principals, and it is reasonable to assume that this trend will continue and worsen (48). Investments in health promotion in schools therefore have a double added value: they not only contribute to the healthy growth of children and adolescents, but also strengthen the attractiveness of the educational profession and promise positive effects on the quality of teaching and education through the promotion of health. However, this requires school principals to consider health promotion not as an additional task, but as an integral part of their educational core mission and their leadership responsibility. In order to achieve this, they need health-promoting leaderships skills that are continuously taught both before they take up their position as a school principal (or deputy principal) and during their principalship. However, the initial examination of preservice and inservice training offers in six European countries carried out here shows that none of the three dimensions of health-promoting leadership has been systematically addressed so far. With this in mind, we see a clear demand:

- for a systematic and in-depth analysis of preservice and inservice training programs for school principals regarding the different facets of health-promoting leadership (self-related health-promoting leadership, staff-related health-promoting leadership, and intervention-related health-promoting leadership),
- to explore the needs for continuous competence development in the area of health promotion and prevention among prospective and existing school principals,
- to develop a European competence framework for health promoting-leadership that takes into account the political and structural characteristics of the educational systems in the European countries,
- to develop and implement a monitoring and evaluation system that provides evidence regarding the effectiveness of health-related training programs for school principals on the implementation of health-promoting interventions in schools, and
- to develop case studies that illustrate how health promotion has been incorporated in preservice and inservice training programs for school leaders that can serve as a practical inspiration and as a basis for mutual learning.

## Data availability statement

The raw data supporting the conclusions of this article will be made available by the authors, without undue reservation.

## Author contributions

KL: Conceptualization, Funding acquisition, Investigation, Resources, Writing—original draft, Writing—review & editing. GG: Resources, Writing—original draft. RR: Formal analysis, Investigation, Resources, Writing—original draft, Writing—review & editing. MS: Formal analysis, Investigation, Resources, Writing—original draft. VV: Formal analysis, Investigation, Resources, Writing—original draft. AS: Formal analysis, Investigation, Resources, Writing—original draft. VS: Formal analysis, Resources, Writing—original draft, Writing—review & editing. TW: Investigation, Resources, Writing—original draft. KD: Conceptualization, Investigation, Resources, Supervision, Writing—original draft, Writing—review & editing.
